# A Metagenomics Pipeline to Characterize Self-Collected Vaginal Microbiome Samples

**DOI:** 10.3390/diagnostics14182039

**Published:** 2024-09-13

**Authors:** Krystal Thomas-White, Evann E. Hilt, Genevieve Olmschenk, Maryann Gong, Caleb D. Phillips, Courtney Jarvis, Nicholas Sanford, Jennifer White, Pita Navarro

**Affiliations:** 1 Evvy, New York, NY 10016, USA; 2 Department of Pathology and Laboratory Medicine, University of Minnesota Medical Center, Minneapolis, MN 55455, USA; 3 Department of Biological Sciences, Texas Tech University, Lubbock, TX 79409, USA; 4 MicrogenDX, Lubbock, TX 79407, USA

**Keywords:** vaginal microbiome, shotgun metagenomic sequencing, vaginitis

## Abstract

Vaginitis is a widespread issue for women worldwide, yet current diagnostic tools are lacking. Bacterial vaginosis (BV) is the most prevalent type of vaginitis, found in 10–50% of reproductive-aged women. Current diagnostic methods for BV rely on clinical criteria, microscopy, or the detection of a few microbes by qPCR. However, many vaginal infections lack a single etiological agent and are characterized by changes in the vaginal microbiome community structure (e.g., BV is defined as a loss of protective lactobacilli resulting in an overgrowth of anaerobic bacteria). Shotgun metagenomic sequencing provides a comprehensive view of all the organisms present in the vaginal microbiome (VMB), allowing for a better understanding of all potential etiologies. Here, we describe a robust VMB metagenomics sequencing test with a sensitivity of 93.1%, a specificity of 90%, a negative predictive value of 93.4%, and a positive predictive value of 89.6% certified by Clinical Laboratory Improvement Amendments (CLIA), the College of American Pathologist (CAP), and the Clinical Laboratory Evaluation Program (CLEP). We sequenced over 7000 human vaginal samples with this pipeline and described general findings and comparisons to US census data.

## 1. Introduction

The vaginal microbiome (VMB) is a complex ecosystem of microorganisms that live inside the vagina. A *Lactobacillus*-dominant microbiome provides protection against infection, while a highly diverse microbiome is often associated with symptoms and vaginal infections [1].

The most common vaginal infection is bacterial vaginosis (BV), a condition that affects up to 29% of women annually [2]. Other conditions of the VMB include vulvovaginal candidiasis (VVC), which will affect 30–50% of women at least once in their lifetime [3], and trichomoniasis, which will affect about 2% of women in the US annually [4].

It has long been understood that vaginal lactobacilli protect against pathogen colonization by producing lactic acid, hydrogen peroxide, and antimicrobial peptides [5]. BV is defined as a loss of lactobacilli and an overgrowth of anaerobic organisms [1]; however, it can also occur alongside infections with yeast or aerobes [6]. The first diagnostic methods for BV relied heavily on symptoms and the detection of lactobacilli by microscopy (i.e., Amsel criteria and Nugent score [7,8]). However, symptom-based diagnosis is often inaccurate because different conditions may cause similar symptoms [9] and co-infections can be common [6]. Additionally, microscopic methods fail to identify the specific organisms present, instead relying on a ratio of rod-shaped bacteria seen with microscopy.

More recently, multiple qPCR panels have been developed for BV diagnosis [10], and while PCR has a very low limit of detection, it can only detect the specific organisms it was designed to detect. Therefore, qPCR misses other potential pathogens that may be contributing to symptoms, as well as the relative amount of protective microbes in the VMB.

Recent research into the VMB has relied on 16S rRNA sequencing due to its relatively low cost and semi-comprehensive assessment of the microbiome [11,12]. Clinical applications of 16S sequencing for bacteria, and ITS for fungi, are also growing in application.

The next advancement in sequencing technology following targeted region amplicon sequencing is shotgun metagenomic sequencing. This method can provide strain-level resolution of all DNA-based microorganisms, including bacteria and yeast from the same assay [13]. Additionally, shotgun sequencing allows for the in-depth study of the function and potential interactions between species as it can identify the genomic differences and pathogenic or biochemical pathways.

Here, we describe the Evvy test, a novel shotgun metagenomics-based test that is approved by the Clinical Laboratory Improvement Amendments (CLIA), the College of American Pathologist (CAP), and the Clinical Laboratory Evaluation Program (CLEP) for VMB profiling that can be used to identify all bacteria and fungi present in a vaginal sample. Additionally, we provide an overview of findings from seven thousand samples, including demographic representation compared to United States (US) census data.

## 2. Materials and Methods

### 2.1. Evvy Test Workflow

**Figure 1:**
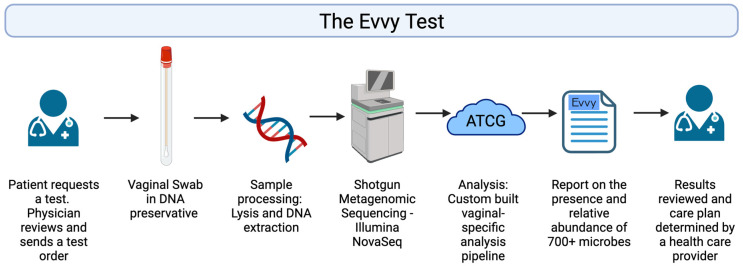
The Evvy VMB test workflow.

An overview of the workflow for Evvy’s VMB laboratory developed test (LDT) is shown in Figure 1. A medical provider submits a lab order for an Evvy vaginal health test. A sample collection kit is shipped directly to the patient. The patient self-collects a vaginal swab and ships the sample at ambient temperature to a CLIA, CAP, and CLEP certified lab (CLIA 45D1086390, CAP 7214171, PFI 9433 Microgen DX, Lubbock, TX, USA). Samples are processed, which includes a chemical and mechanical lysis, host depletion, and DNA extraction using an automated extraction handling instrument. NGS libraries are prepared, multiplexed, quality checked, and sequenced on the Illumina NovaSeq 600 (Illumina, San Diego, CA, USA). Sequencing data are processed through Evvy’s pipeline, which is specifically designed to characterize the vaginal microbiome. Samples that pass detection thresholds are reported to the provider and patient.

### 2.2. Isolate Sequencing

A total of 168 isolates were included in the sensitivity and specificity study. Isolates were ordered from ATCC (Supplementary Table S1) and spiked into collection tubes containing DNA preservatives. All samples were processed using the shotgun metagenomic sample prep described below. Sensitivity, specificity, positive, and negative predictive values were calculated as previously described [14]. Samples with classifications that matched (>90% of mapped reads) the input ATCC strains were considered true positives. False positives include ATCC species that were identified for other ATCC isolates not matching the given species. The test set also included true negatives, including ATCC strains that were not represented in the classification database, as well as molecular-grade water controls. False negative samples include 12 samples that were sequenced below the 20,000 read sequencing threshold.

### 2.3. Limit of Detection and Precision Studies

The limit of detection and precision studies were carried out with a Gram-negative organism (*Klebsialle oxytoca*), a Gram-positive organism (*Lactobacillus gasseri*), and a fungal species (*Candida albicans*) available through ATCC. On each of 3 days, 8 ten-fold dilutions were prepared and run through the protocol as 3 technical replicates. The limit of detection (LOD) was determined as the concentration of cells that could be detected in ≥95% of samples tested with a coefficient of variation ≤5%.

Inter-assay and intra-assay reproducibility (precision) were demonstrated by comparing the average relative abundance (the reported value) within and between runs over three days of testing by two different technicians. To be considered reproducible, the coefficient of variation of the average relative abundance must be less than 5% for each day of testing (intra-assay reproducibility) and less than 5% over 3 days of testing (inter-assay reproducibility) at the established LOD.

### 2.4. Mock Community Analysis

Whole-cell vaginal mock communities were purchased from ATCC (MSA-2007), containing equal amounts of *Gardnerella vaginalis*, *Lactobacillus gasseri*, *Mycoplasma hominis*, *Prevotella bivia*, *Streptococcus agalactiae*, and *Lactobacillus jensenii*. Prior to DNA extraction, Hep2 cells (CCL-23) were spiked in to mimic the host DNA seen within a vaginal sample. The mock communities plus host were serially diluted (1:10), then processed through the normal Evvy pipeline. The undiluted samples went through an in silico dilution process, where the undiluted sample was subsampled to 1–4-log fewer sequence reads (from 1.6 million reads to 160 reads).

### 2.5. Study Participants, Ethics, and Sample Collection and Transportation

Clinical samples were obtained from participants on the Evvy platform in the US. All study participants consented to participate in the study, and all study procedures were approved by a federally accredited Institutional Review Board (IRB# 20220118.evvy). Participants represented varying ages and ethnic groups.

Patients self-collected vaginal swabs and placed them into Copan eNAT collection tubes (Copan, Murrieta, CA, USA); samples were shipped to the laboratory (Microgen DX, Lubbock, TX, USA) and processed through a CLIA/CAP/CLEP certified shotgun metagenomics pipeline within 5 days. Each participant completed a questionnaire which includes symptoms, related diagnoses, and demographic information.

### 2.6. Shotgun Metagenomics Analysis of Vaginal Samples

For the shotgun metagenomics analysis, a validated pipeline was used. Vaginal samples underwent host depletion with PMA [15], chemical lysis using metapolyzyme (Sigma, St. Louis, MA, USA), followed by bead beating and DNA extraction on an automated extraction instrument (KingFisher FLEX, Temecula, CA, USA, Thermo Scientific, Waltham, MA, USA). Each extraction batch includes a negative control (NC, water) and a mock community positive control (PC-ATCC MSA-2007).

Evvy’s bioinformatics platform includes quality control, host depletion, and taxonomic profiling. Trimmomatic [16] was used to trim and filter raw reads to remove low quality bases. Following quality filtering, remaining data are further refined by eliminating human DNA sequences by mapping to the latest human reference (GRCh38). Remaining reads are mapped to a proprietary database of precomputed genomic signatures for species-level classification. Only classifications with >0.75% relative abundance are included in reports.

The unique signatures comprising the database are computed from microbial whole genomes isolated from the urogenital tract and contain more than 4000 microbial genomes, representing 700 bacterial species. Between January and March 2023, genomes were collected from publicly available repositories (NCBI and EBI) with any isolate documented to be collected from “urogenital” or “vaginal” sources. To accurately represent the phylogenetic diversity of underrepresented taxa, some genomes were included from other human microbiome sites. Public repositories are incomplete in their representation of the vaginal microbiome; for this reason, we also included metagenome-assembled genomes generated from Evvy test data.

## 3. Results

### 3.1. Sensitivity and Specificity

The sensitivity and specificity study included a total of 162 ATCC strains (Supplementary Table S1) plus 50 16S negative patient vaginal samples spiked with molecular-grade water (total of 202 samples). Samples must have at least 20K reads. Any sample that had greater than 50% relative abundance and greater than 90% identity was considered a positive. Any sample that did not meet those thresholds was considered a negative. True positives were samples with accurate species classification, while false positives were samples that passed all thresholds but had inaccurate species classification. We note that 8/11 false positives were accurate at the genus level (Supplementary Table S1). True negatives were samples that did not meet the thresholds because they were not included in the database; therefore, it is expected that they do not produce any accurately identified mapped reads. False negatives failed threshold requirements even though they were in the database. We note that two-sevenths of false negatives had accurate overall classification, and four of the remaining five were accurate at the genus level even though the samples did not meet thresholding requirements (Supplementary Table S1).

Ninety-five samples were true positives with data from ATCC and the NovaSeq6000 (Illumina, San Diego, CA, USA) matching. Forty-nine isolates were not included in the reference database and therefore produced true negative results, plus the fifty negative patient samples spiked with water. The test therefore had a measured sensitivity of 93.1%, a specificity of 90%, a negative predictive value (NPV) of 93.4%, and a positive predictive value (PPV) of 89.6% (Table 1).

### 3.2. Limit of Detection and Precision

The bacterial LOD was determined to be 3000–1640 CFU/mL, and the fungal LOD was determined to be 2400 CFU/mL. The fungal species could be detected at 240 CFU/mL; however, the inter-assay reproducibility was unacceptable at this concentration (CV% = 13.732) (Supplementary Table S2).

All species included in the inter- and intra-assay reproducibility study were reproducible within runs at the assay limit of detection over the three testing days of the study (Supplementary Table S2).

### 3.3. Mock Community Analysis

**Figure 2:**
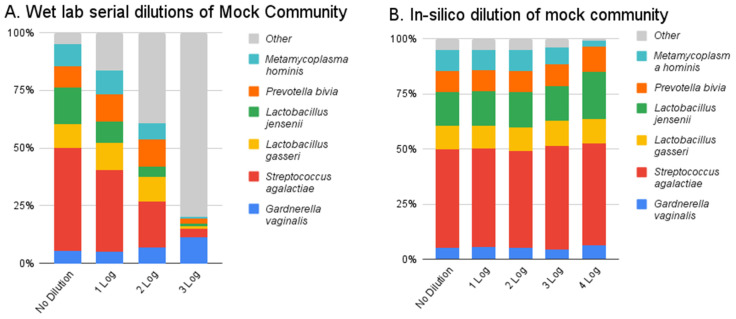
Shotgun metagenomics resolution of mock community samples: (**A**) Of 1:10 dilution of mock community performed in the wet lab and (**B**) an in-silico subsampling of the initial mock community sample down to a 4-log decrease.

Wet lab serial dilutions of mock community plus host cells show a stepwise decrease in the detection of the mock community as the amount of DNA decreases (Figure 2A). This experiment was completed in duplicate, and 1 representative is shown in Figure 2. An expected corresponding amount of “kit-ome” is detected [17], listed here as “other”. All components of the mock community could be detected at the third dilution, which reached the sequencing limit of detection of twenty thousand reads.

An in-silico subsampling of the initial mock community sample shows that our algorithm is accurate at detecting all components of the mock community at read levels ranging from 1.6 million reads (no dilution) to 160 reads (4 log decrease) (Figure 2B).

#### 3.3.1. Findings from Evvy’s Vaginal Metagenomics Test

**Figure 3:**
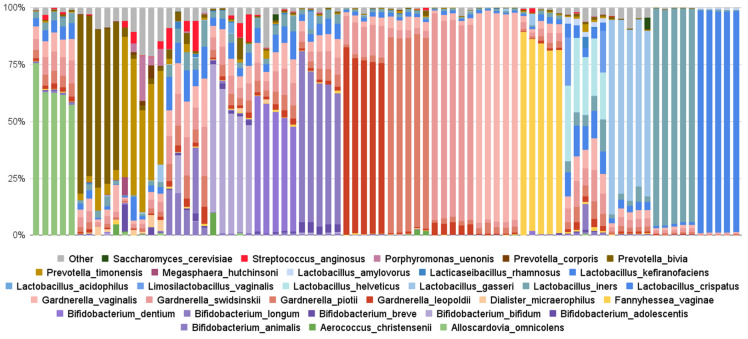
Relative abundance graphs of vaginal samples. These profiles are example profiles containing the top 10 species detected by frequency and relative abundance (Table 2).

Using Evvy’s sampling and taxonomy classification pipeline, a total of 7158 samples have been sequenced in 4.5 months. Of those samples, 178 microbial species have been identified above the detection threshold. The top ten species most commonly detected include all four named *Gardnerellas* (*G. vaginalis*, *G. swidinskii*, *G. piotii*, *G. leopoldii*), *Lactobacillus iners*, *L. crispatus*, *Fannyhessae* (previously *Atopobium*) *vaginae*, *Bifidobacterium animalis*, *Prevotella bivia*, and *P. timonensis* (Table 2). The top ten species with the highest average abundance include *Lactobacillus* and *Bifidobacterium* species, two *Gardnerella* species, and *Alloscardovia omnicolens* (Table 2). To illustrate the community structure in samples containing these commonly found organisms, we pulled five random samples for each of the most abundant species (Figure 3). We see that some samples are not diverse and consist primarily of a single taxon (i.e., *L. crispatus*, *L. gasseri*, or *Gardnerella* dominant), while other samples are more diverse (i.e., *L. helveticus*, *Prevotella*, and *Gardnerella* non-dominant samples Figure 3).

#### 3.3.2. Co-Occurrence of Pathogens Detected

Shotgun metagenomics can detect a variety of bacteria and yeast, allowing for the accurate identification of all potential bacterial pathogens. Using the Evvy test, multiple potential pathogens can be identified within each sample. Across 7158 samples, *Candida* species (*C. albicans*, *C. glabrata*, and *C. parapsilosis*) rarely co-occurred, but were commonly found alongside both anaerobic, BV-associated organisms, like *Prevotella*, *Mobiluncus*, *Megasphaera*, and *Gardnerella* (Supplementary Table S3). Aerobic organisms (specifically, *E. coli*, *Klebsiella*, *E. faecalis*, and Group B Strep) also commonly co-occurred with BV-associated organisms (Table S3).

#### 3.3.3. Comparison to US Census Data

**Figure 4:**
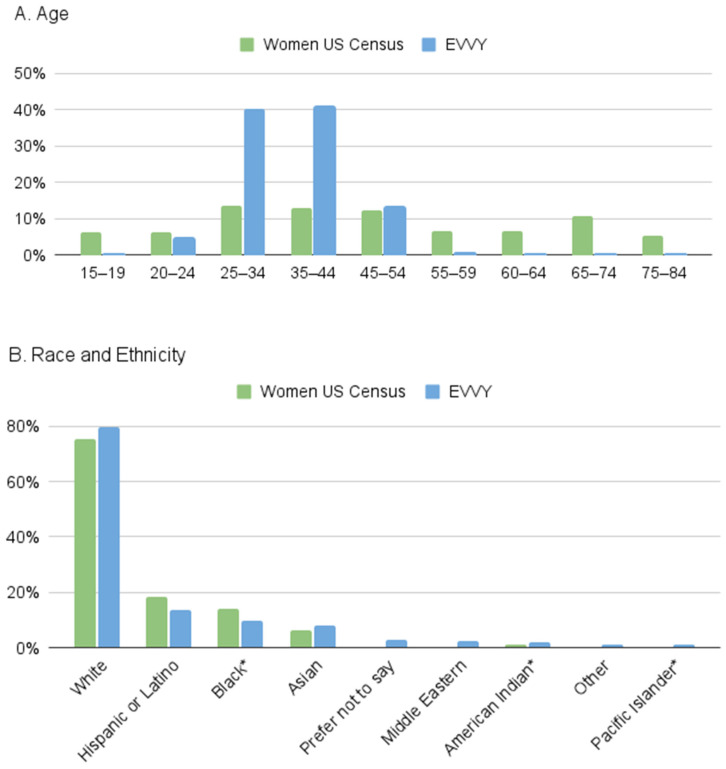
Comparison of Evvy’s user data to the 2020 US census data. Percent of samples stratified by (**A**) age, or (**B**) self-reported race and ethnicity compared to reported US census data. * The follow race/ethnicity options were shortened for this figure: Black or African American, American Indian or Alaskan Native, Native Hawaiian or Pacific Islander.

A comparison of the 7158 samples to the US census data shows that Evvy users tend to be younger (aged 20–44) than the US population as a whole, and fewer Evvy users are above age 55 (Figure 4A). Evvy samples closely match the distributions of race and ethnicity across the US, with similar proportions of Hispanic or Latino, Black or African American, Asian, and Native American or Alaskan Natives (Figure 4B).

## 4. Discussion

In this report, we describe the validation of the first CLIA/CAP/CLEP certified shotgun metagenomic test for vaginal microbiome (VMB) samples. The Evvy test includes patient-collected vaginal swabs, complete sample lysis, DNA extraction, sequencing library preparation, NovaSeq sequencing, and a bioinformatic analysis platform that includes taxonomic classification to the species level.

This metagenomic test provides a comprehensive view of the microbiome that can be used for patient diagnoses, increasing access to care through self-sampling at home, and future research into the VMB.

Vaginitis affects 29% of women annually [2] and is one of the primary reasons women seek medical care [18]. Yet the average wait time for an OB-GYN appointment is 31 days [19]. The Evvy test can expand access to care by providing the ability to self-test, receive lab results, and access physician-lead care from home.

Studies show that vaginitis patients are more likely to be misdiagnosed than correctly diagnosed by a doctor [9]. The Evvy pipeline demonstrates the accurate detection of vaginal isolates with a sensitivity of 93.1%, a specificity of 90%, a negative predictive value (NPV) of 93.4%, and a positive predictive value (PPV) of 89.6%. These numbers are above the acceptable range for a clinical metagenomics test [20,21] and allow for the accurate characterization of the vaginal microbiome.

Vaginitis remains poorly understood [22], and symptoms can often be the result of mixed infections [6]. A co-occurrence analysis showed that different *Candida* species were found to rarely co-occur together. But *Candida* did co-occur with BV organisms as well as a variety of aerobic organisms (*E. coli*, *Klebsiella*, *E. faecalis*, and Group B Strep). Previous work from Evvy’s group also identified that when aerobic organisms are present, symptoms tend to be more severe [23]. PCR tests only detect a small panel of organisms; for example, no currently available PCR panel detects anaerobic organisms or all *Candida* species. This illustrates how the Evvy test allows for a broader characterization of the microorganisms present in vaginal samples, enabling a provider to evaluate multiple etiologies with one test.

One limitation of the Evvy test is the turnaround time from sampling to metagenomic sequencing results (about 12 days). Therefore, the Evvy test may not be the appropriate test for every patient. PCR may be a more appropriate choice when physicians are looking for the presence of specific low-abundance pathogens. While shotgun metagenomics allows for a breadth of organism detection, PCR still has a much lower limit of detection than any current next generation sequencing (NGS) technology available. However, for patients with negative PCR tests, a history of recurrent infections, or suspected co-infections, shotgun metagenomic sequencing of the VMB can be useful for informing diagnosis and treatment. Providers looking for a more comprehensive view of the VMB may also leverage this type of test.

Moreover, recent research has highlighted the relationship between VMB dysbiosis and a wide array of gynecological and obstetric conditions [12,24,25,26,27,28,29,30]. Through this testing platform, Evvy has generated the largest mNGS vaginal microbiome and health profile dataset ever collected. Evvy’s platform includes patients from all fifty states and very closely matches 2020 US census data for race and ethnicity. To our knowledge, no other dataset has included both the large sample sizes as well as a breadth of racial and demographic representation. Its wide representation means that the Evvy’s multi-dimensional dataset can be used to better understand how the VMB changes in response to treatments, life events (pregnancy, menopause, etc.), and in association with different gynecological diseases. These analyses will be described in upcoming publications.

In conclusion, the Evvy test represents a significant advancement in the diagnosis and understanding of vaginitis. Its capability to provide a more comprehensive view of the VMB opens new avenues for personalized medicine and enhances our understanding of the microbiome’s role in female health.

## 5. Conclusions

Here, we report a new CLIA/CAP/CLEP certified laboratory developed test that comprehensively and accurately identifies organisms in the vaginal microbiome. The test has a sensitivity of 93.1%, a specificity of 90%, a negative predictive value (NPV) of 93.4%, and a positive predictive value (PPV) of 89.6%. An analysis of seven thousand results of the Evvy test shows a high co-occurrence of potential pathogenic organisms and racial representation similar to the US population. This test provides a more comprehensive characterization of the composition of the whole vaginal microbiome with one swab.

## Figures and Tables

**Table 1:** Sensitivity and specificity based on sequencing 168 ATCC isolates ATCC.

		Positive	Negative	Total	Predictive Value
NovaSeq	**Positive**	95	11	106	89.6% (95/106) PPV
	**Negative**	7	99	106	93.4% (99/106) NPV
	**Total**	102	110		
		Sensitivity 93.1% (95/102)	Specificity 90% (99/110)		

**Table 2:** The top 10 species and genera identified in 7158 human vaginal samples, based on their prevalence.

Top 10 Species by Frequency of Detection	Percentage of Tests Detected	Top 10 Species by Relative Abundance	Average Abundance	Percent of Tests Detected
*Gardnerella vaginalis*	99%	*Lactobacillus crispatus*	33%	93%
*Gardnerella swidinskii*	99%	*Lactobacillus helveticus*	23%	0.1%
*Lactobacillus iners*	95%	*Bifidobacterium dentium*	19%	0.9%
*Gardnerella piotii*	95%	*Lactobacillus iners*	18%	95%
*Lactobacillus crispatus*	93%	*Lactobacillus gasseri*	15%	14%
*Gardnerella leopoldii*	78%	*Bifidobacterium longum*	13%	2%
*Prevotella bivia*	46%	*Bifidobacterium bifidum*	13%	0.7%
*Fannyhessae vaginae*	33%	*Gardnerella swidinskii*	12%	99%
*Bifidobacterium animalis*	31%	*Alloscardovia omnicolens*	12%	2%
*Prevotella timonensis*	27%	*Gardnerella vaginalis*	11%	99%

## Data Availability

This research was sponsored by Evvy and the authors of the paper who have access to the data are employees or scientific collaborators of Evvy who have signed contracts with Evvy to be bound by Evvy’s privacy policy and access restrictions. Additional data can be made available through a Data Transfer Agreement that protects the privacy of participants’ data; interested researchers may make requests by contacting kthomasw@evvy.com. The information provided by interested researchers will be used to generate a Data Transfer Agreement (DTA). The DTA protects the privacy of the participants’ data and will need to be signed by both the institution and Evvy before data can be transferred.

## Supplementary Materials

The following supporting information can be downloaded at https://www.mdpi.com/article/10.3390/diagnostics14182039/s1, Table S1, S2, and S3.
